# Prognostic value of normal sodium levels in patients with metastatic renal cell carcinoma receiving tyrosine kinase inhibitors

**DOI:** 10.3389/fonc.2022.918413

**Published:** 2022-08-16

**Authors:** Giandomenico Roviello, Martina Catalano, Ugo De Giorgi, Marco Maruzzo, Sebastiano Buti, Elisabetta Gambale, Giuseppe Procopio, Carlotta Ottanelli, Enrico Caliman, Luca Isella, Pierangela Sepe, Nicole Brighi, Matteo Santoni, Luca Galli, Raffaele Conca, Laura Doni, Lorenzo Antonuzzo

**Affiliations:** ^1^Department of Health Sciences, University of Florence, Florence, Italy; ^2^Department of Oncology, Istituto di Ricovero e Cura a Carattere Scientifico (IRCCS) Istituto Romagnolo per lo Studio dei Tumori (IRST) “Dino Amadori”, Meldola, Italy; ^3^Oncology Unit 1, Istituto Oncologico Veneto IOV - Istituto di Ricovero e Cura a Carattere Scientifico (IRCCS), Padua, Italy; ^4^Department of Medicine and Surgery, University of Parma, Parma, Italy; ^5^Medical Oncology Unit, University Hospital of Parma, Parma, Italy; ^6^Clinical Oncology Unit, Careggi University Hospital, Florence, Italy; ^7^Department of Medical Oncology, Fondazione Istituto di Ricovero e Cura a Carattere Scientifico (IRCCS) Istituto Nazionale dei Tumori di Milano, Milan, Italy; ^8^Department of Experimental and Clinical Medicine, University of Florence, Florence, Italy; ^9^Oncology Unit, Macerata Hospital, Macerata, Italy; ^10^Medical Oncology Unit 2, Azienda Ospedaliero-Universitaria Pisana, Pisa, Italy; ^11^Unit of Medical Oncology, Department of Onco-Hematology, Centro di Riferimento Oncologico della Basilicata (IRCCS-CROB), Rionero in Vulture, Italy; ^12^Medical Oncology Unit, Careggi University Hospital, Florence, Italy

**Keywords:** metastatic RCC, serum sodium, prognostic factors, PFS, OS

## Abstract

**Background:**

Although serum sodium concentration, particularly hyponatremia, has been shown to be a prognostic marker of survival in metastatic renal cell carcinoma (mRCC), the impact of normal sodium levels has not been investigated. Herein, we investigate the influence of normonatremia in mRCC patients treated with tyrosine kinase inhibitors (TKIs).

**Materials and methods:**

For this retrospective study, the clinical and biochemical data of patients treated with first-line TKIs for mRCC were available from seven Italian cancer centers. We collected natremia levels at baseline and first evaluation after treatment excluding patients with sodium levels outside the normal range (<135 or >145 mEq/L). The remaining patients were subdivided into two groups according to the median sodium value: natremia patients with <140 mEq/L (*n* = 132) and baseline natremia patients with ≥140 mEq/L (*n* = 185). Subsequently, we analyzed the impact of sodium levels on response rate (RR), disease control rate (DCR), progression-free survival (PFS), and overall survival (OS). PFS and OS were estimated through the Kaplan–Meier method, and differences between groups were examined by the log-rank test. Univariate and multivariate Cox regression analyses were applied to evaluate the prognostic factors for PFS and OS.

**Results:**

Of the 368 patients, 317 were included in the analysis, 73.1% were men, and the median age was 67 years (range 36–89). When comparing patients with baseline natremia ≥140 mEq/L (*n* = 185) to patients with natremia <140 mEq/L (*n* = 132), the PFS was 15 vs. 10 months (*p* < 0.01) and the OS was 63 vs. 36 months, respectively (*p* = 0.02). On the first evaluation, patients with serum sodium ≥140 mEq/L had longer PFS (15 vs. 10 months, *p* < 0.01) and OS (70 vs. 32 months, *p* < 0.01) than patients with levels <140 mEq/L. Moreover, clinical outcomes showed a significant improvement in patients with natremia ≥140 mEq/L compared with patients with levels <140 mEq/L both at baseline and first evaluation: PFS was 19 vs. 11 months (*p* < 0.01) and OS was 70 vs. 36 months (*p* < 0.01), respectively.

**Conclusions:**

To the best of our knowledge, this is the first study to investigate the impact of normonatremia in mRCC. We found that serum sodium levels <140 mEq/L at baseline and first assessment are independently associated with worse PFS and OS in mRCC patients treated with TKIs in the first-line setting.

## 1 Introduction

Renal cell carcinoma represents approximately 3% of all malignancies in adulthood, accounting for approximately 430,000 new cases and 179,368 deaths in 2020 worldwide (1). Over the last decades, the treatment of metastatic RCC (mRCC) has dramatically changed, including both immunotherapeutic and multitargeted receptor tyrosine kinase drugs, with progressive prognosis improvement. Despite the different treatment options available to date, some patients continue to be unresponsive to systemic treatments or rapidly progress. The selection of optimal treatment is based on prognostic models including clinical characteristics and biochemical examination. The Memorial Sloan Kettering Cancer Center (MSKCC) and the International Metastatic RCC Database Consortium (IMDC) risk score classifications are widely used in clinical practice and stratify patients into three risk groups: favorable, intermediate, or poor (2, 3). Nowadays, the optimal therapy for mRCC is generally chosen using this stratification, but examination of potentially new predictive and prognostic markers is highly warranted. Serum sodium levels have been shown to be a prognostic marker in several diseases, including congestive heart failure, liver cirrhosis, systemic infections, and malignancies (4–9). Hyponatremia, commonly defined as a serum sodium level lower than 135 mEq/L, is an independent prognostic factor for solid malignancies such as hepatocellular carcinoma (10), advanced small cell lung cancer (11, 12), advanced gastric cancer (13), and localized RCC (14). In mRCC patients treated with vascular endothelium growth factor (VEGF)- and mammalian target of rapamycin (mTOR)-targeted agents, hyponatremia has been correlated with a worse prognosis (15). It is also a negative predictive factor for cancer-specific survival in mRCC patients treated with first-line immunotherapy (interleukin-2 and interferon-α) or molecular targeted therapy (16). The mechanisms underlying the development of hyponatremia in RCC patients still remain unclear. An ectopic inappropriate production of antidiuretic hormone (ADH), although uncommon in patients with RCC compared with other tumor types (e.g., lung, head and neck, and breast cancers) or post-nephrectomy renal dysfunction, may partly explain hyponatremia (12, 14). Although serum sodium levels are routinely measured at baseline and during cancer treatment, the role of normonatremia in mRCC has not been investigated. Herein, we perform a multicenter retrospective study to evaluate the potential correlation between normonatremia value and outcomes in mRCC patients treated with first-line therapy of tyrosine kinase inhibitors (TKIs).

## 2 Materials and methods

### 2.1 Patients and treatment

The clinical data for all consecutive mRCC patients receiving first-line treatment with TKIs from January 2010 to December 2017 at seven Italian Oncology Centers were retrospectively analyzed. The inclusion criteria were as follows: patients aged ≥18 years, histologically confirmed mRCC, at least one cycle of treatment completed, and values of serum sodium available at baseline and after one cycle of therapy. Demographic data, histological type, Eastern Cooperative Oncology Group-Performance Status (ECOG-PS), number of metastatic sites, risk group according to the IMDC criteria, TKI used as first-line treatment, and serum sodium values were recorded for all patients. Normal thyroid function, namely, thyroid-stimulating hormone between 0.5 and 5.0 mU/L and free thyroxine 4 between 0.7 and 1.9 ng/dl, was also considered necessary. Patients with outranging serum sodium values (<135 or >145 mEq/L) were excluded as well as those suffering from any serious cardiovascular conditions (i.e., myocardial infarction, ejection fraction <40%, uncontrolled blood pressure, or previous thromboembolism). The initial dose of TKIs was chosen according to the European Medicines Agency guidelines: sunitinib was administered 50 mg orally once a day for 4 consecutive weeks followed by 2 weeks of rest (scheme 4 on/2 off), and pazopanib 800 mg and cabozantinib 60 mg were administered once daily in all cases until disease progression or unacceptable toxicity. Dose adjustment was performed according to the data sheet. This study was approved by the local ethics committee (Tuscany section AREA VASTA CENTRO, number: 14565_oss) and written informed consent was obtained from all patients.

### 2.2 Assessment

The serum sodium, as a routine laboratory assessment, was performed at baseline (within 10 days of starting the treatment) and prior to any therapy cycles. The first assessment was performed approximately 40 days after initiation of sunitinib and 26 days after initiation of pazopanib and cabozantinib. Normonatremia was defined as serum sodium values between 135 and 145 mEq/L. Blood pressure, proteinuria, and thyroid function were monitored during treatment. Response evaluation was performed every 3 months by spiral computed tomography and evaluated according to the Response Evaluation Criteria in Solid Tumor (RECIST) version 1.1 (17). Efficacy was evaluated as overall survival (OS) and progression-free survival (PFS). Adverse events (AEs) during TKI administration were monitored by the investigators and reported. Treatment-related AEs were assessed using the Common Terminology Criteria of Adverse Events (CTCAE) version 4.0 (18). Variables considered for prognostic correlations included age, sex, histology, previous surgery, ECOG-PS, IMDC score, number of metastatic sites, and basal and first evaluation levels of serum sodium.

### 2.3 Outcome variables

This study aimed to evaluate whether normal sodium levels (range 135–145 mEq/L) correlate with the efficacy and survival of patients with metastatic RCC treated with TKI as first-line treatment. For this purpose, patients were divided into two groups based on the serum sodium values: above and equal to or below the median value (140 mEq/L; reference range, 135–145 mmol/L). Primary outcomes were PFS and OS; secondary endpoints were response rate (RR) and disease control rate (DCR). PFS was defined as the time from the date of enrollment to disease progression or death. OS was defined as the time from registration until death from any cause. Disease control rate was defined as the proportion of patients with the best overall response, taken as complete response (CR), partial response (PR), or stable disease (SD), while RR was defined as the proportion of patients showing CR or PR.

### 2.4 Statistical analysis

The appropriate descriptive statistics were used for demographics and tumor features. Continuous variables were expressed as median and range (minimum and maximum values), while categorical variables were presented as number and percentage. PFS and OS were estimated with the Kaplan–Meier method, and differences between groups were compared with the log-rank test. The estimated hazard ratios (HRs) and their two-sided 95% confidential interval (CI) were calculated using the Cox proportional hazard model. After the univariate analysis to evaluate the prognostic factors for PFS and OS, parameters with *p ≤*0.05 were considered statistically significant and included in the multivariate analysis. A multivariate Cox regression model, evaluating IMDC score, ECOG-PS, and previous nephrectomy, as well as serum sodium at baseline and first assessment, was used to adjust for these potential confounding factors. For the survival analysis, variables were dichotomized and the dichotomized value of serum sodium was correlated with the clinical and biochemical variables using Fisher’s exact test. Statistical analyses were performed using Stata version 9.1.

## 3. Results

### 3.1 Patient characteristics

#### 3.1.1 Patient features

**Figure 1:**
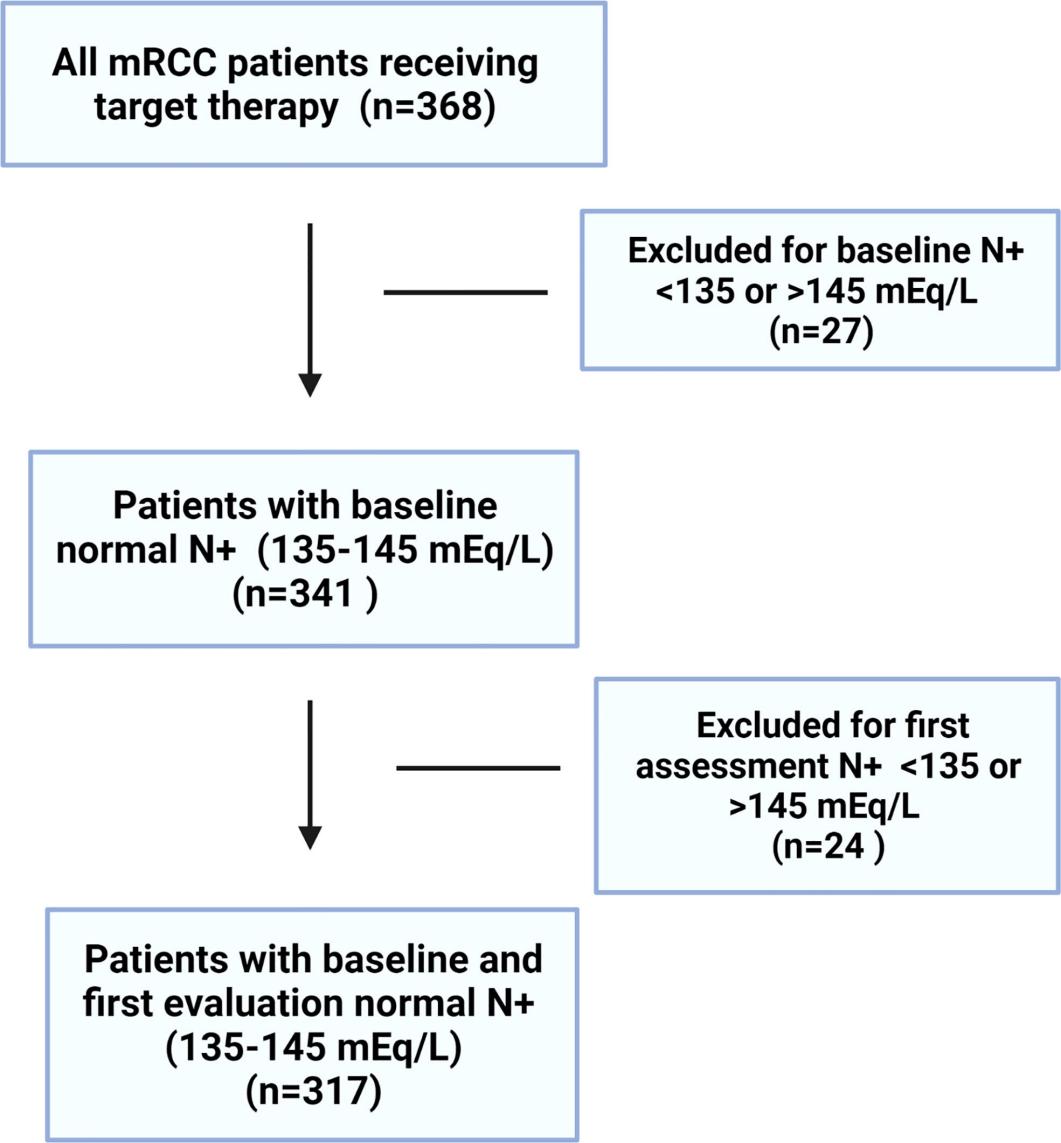
The study flowchart.

From January 2010 to December 2017, 368 patients diagnosed with metastatic RCC and treated with first-line TKI were retrospectively considered. Twenty-seven patients at baseline and 24 patients at first evaluation had serum sodium outside the limits of our laboratory reference range (<135 or >145 mEq/L) and were consequently excluded. Overall, 317 patients were considered for analysis (**Figure 1**).

The median follow-up was 33 months (range 2–141). The median age of the cohort was 67 (range 36–89) years and 232 (73.1%) were men. The majority of the patients had clear cell histology (86.7%) with a prevalence of intermediate–poor IMDC risk (66.5%). Less than half of the patients showed ECOG-PS ≥1, and 95 (29.9%) patients presented three or more metastatic sites. The patients received one of the following TKIs: sunitinib (54.9%), pazopanib (34.7%), or cabozantinib (10.4%). Overall, 271 (85.4%) patients had undergone surgical treatment and 97 (30.6%) had received more than one line of therapy after TKI. All baseline patient characteristics are summarized in **Table 1**.

**Table 1:** Patients’ baseline characteristics.

	All patients (*N* = 317)
**Age**	
Median (range)	67 (36–89)
**Gender**, *n* (%)	
Male	232 (73.1)
**Histology**, *n* (%)	
Clear cell RCC	275 (86.7)
**Previous nephrectomy,** *n* (%)	
Yes	271 (85.4)
**ECOG,** *n* (%)	
≥1	152 (47.9)
**IMDC score,** *n* (%)	
Intermediate–poor	211 (66.5)
**Number of metastatic sites,** *n* (%)	
≥3	95 (29.9)
**First-line therapy,** *n* (%)	
Sunitinib Pazopanib Cabozantinib	174 (54.9) 110 (34.7) 33 (10.4)
**Line of therapy after TKI,** *n* (%)	
>1	97 (30.6%)
**Pre-treatment Na+** (mEq/L)	
Median (range)	140 (135–145)
**First evaluation Na+** (mEq/L)	
Median (range)	140 (135–145)

RCC, renal cell carcinoma; IMDC, international Metastatic Renal Cell Carcinoma Database Consortium; TKI, tyrosine kinase inhibitor.

#### 3.1.2 Patient baseline features according to natremia

Patient baseline characteristics according to pre-treatment and first evaluation natremia (<140 or ≥140 mEq/L) are reported in **Tables S1–3**. At pre-treatment evaluation, 185 (58.3%) patients had natremia ≥140 mEq/L (median 141, range 140–145), whereas 132 (41.7%) had <140 mEq/L (median 138, range 135–139). There were no statistically significant differences in demographic and clinical features between patients with serum sodium <140 mEq/L and those with ≥140 mEq/L at pre-treatment evaluation.

On first assessment, 184 (58%) patients showed serum sodium ≥140 mEq/L (median 141, range 140–145 mEq/L) and 133 (42%) showed <140 mEq/L (median 138, range 135–139 mEq/L). Likewise, at pre-treatment evaluation, no other statistically significant differences were recorded. Features of patients with serum sodium ≥140 mEq/L at both baseline and first assessment did not differ from those of patients with sodium values <140 mEq/L before treatment or at first evaluation.

### 3.2 Efficacy outcomes and best response

Patient survival was evaluated according to previously reported serum sodium profiles (<140 or ≥140 mEq/L) and assessment time (baseline and at first evaluation from the start of treatment).

**Figure 2:**
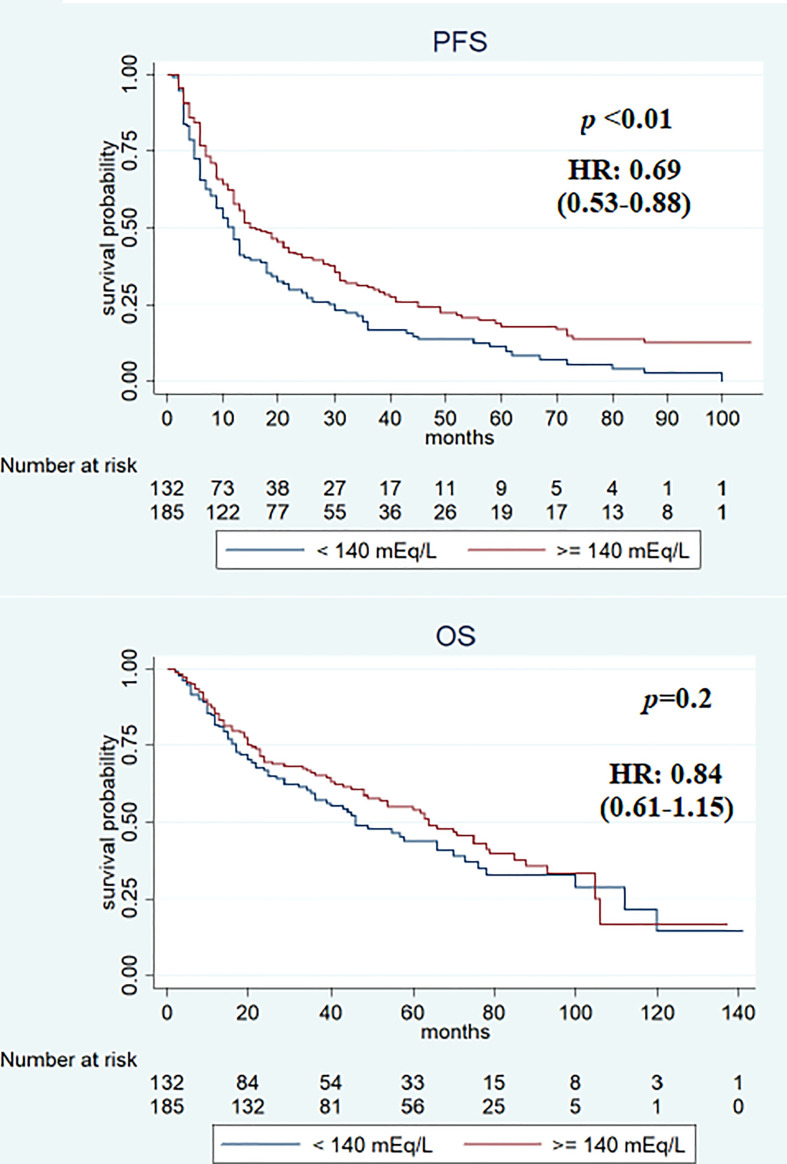
Kaplan–Meier survival estimate according to baseline sodium serum values.

**Figure 3:**
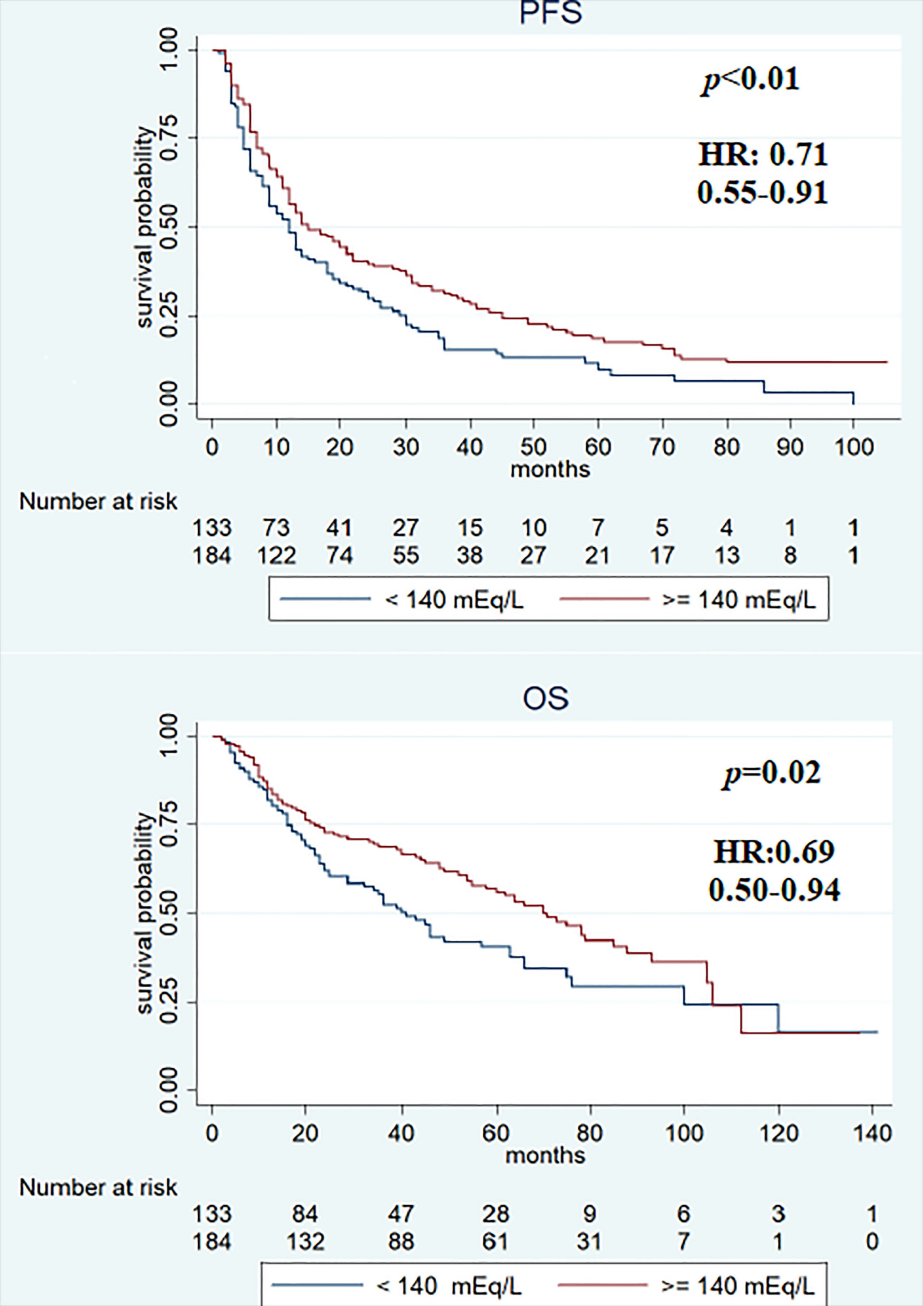
Kaplan–Meier survival estimate according to first assessment after treatment with different sodium serum values.

**Figure 4:**
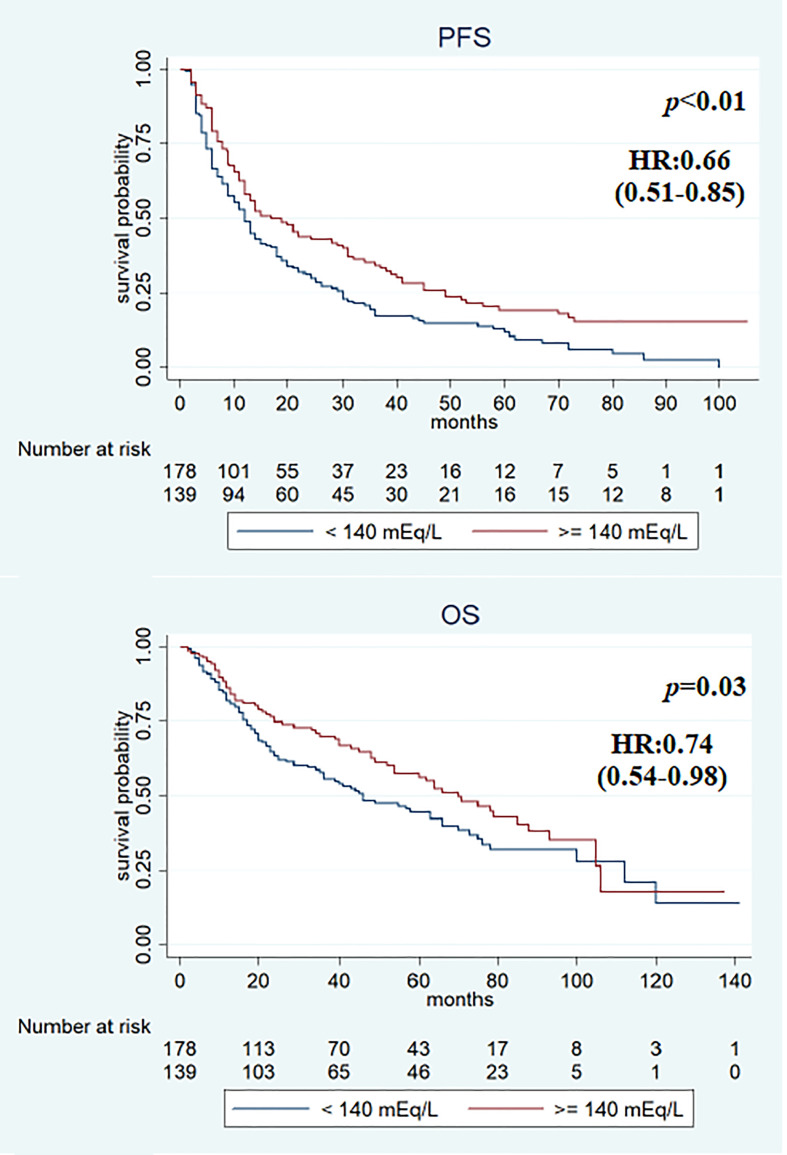
Kaplan–Meier survival estimate according to baseline and first assessment with sodium serum ≥140 mEq/L (vs. at least sodium value <140 mEq/L).

Median PFS was significantly longer in the cohort with high (≥140 mEq/L) than in low (<140 mEq/L) pre-treatment sodium levels (16 and 12 months, respectively, *p* < 0.01). On the other hand, no significant differences were observed when comparing the two cohorts (high vs. low) for median OS (64 vs. 46 months; *p* = 0.2) (**Figure 2**), RR (38.4% vs. 43.2%, *p* = 0.2), and DCR (85.9% vs. 79.5%, *p* = 0.1). On first evaluation, patients with serum sodium ≥140 mEq/L (*n* = 184) had longer median PFS and OS compared with patients with <140 mEq/L (*n* = 133) [15 vs. 12 months (*p* < 0.01) and 70 vs. 41 months (*p* = 0.02), respectively] (**Figure 3**). No differences were observed in RR (40.2% vs. 40.6%; *p* = 0.9) and DCR (85.8 vs. 79.6%; *p* = 0.1) between high and low sodium levels. Moreover, a significant improvement in clinical outcome was seen in patients with natremia ≥140 mEq/L both at baseline and first evaluation (*n* = 139) (median PFS was 19 months and median OS was 70 months) (*p* < 0.01) compared to patients with natremia <140 mEq/L (*n* = 178) (median PFS was 12 months and median OS was 46 months) (*p* = 0.03) (**Figure 4**). The difference between RR and DCR remained non-statistically significant (*p* = 0.2 and *p* = 0.1, respectively) (**Table 2**).

**Table 2:** Best response, PFS, and OS according to serum sodium values.

	RR, *n* (%)	DCR, *n* (%)	Median PFS, months (95% CI)	Median OS, months (95% CI)
**All patients** (*N* = 317)	128 (40.4)	264 (83.3)	13 (12–18)	60 (46–70)
Pre-treatment Na+, (*n*)				
≥140 mEq/L (185)	71 (38.4)	159 (85.9)	16 (13–22)	64 (49–79)
<140 mEq/L (132)	57 (43.2)	105 (79.5)	12 (9–13)	46 (36–70)
	*p* = 0.2	*p* = 0.1	*p *< 0.01	*p* = 0.2
**First evaluation Na+**, (*n*)				
≥140 mEq/L (184)	74 (40.2)	158 (85.8)	15 (12–21)	70 (55–85)
<140 mEq/L (133)	54 (40.6)	106 (79.6)	12 (9–15)	41 (29–57)
	*p* = 0.9	*p* = 0.1	*p *< 0.01	*p* = 0.02
**Pre- and first Na+**, (*n*)				
≥140 mEq/L (139) <140 mEq/L (178)	51 (36.6) 77 (43.2)	121 (87.0) 143 (80.3)	19 (12–29) 12 (9–15)	70 (54–88) 46 (35–66)
	*p* = 0.2	*p* = 0.1	*p* < 0.01	*p* = 0.03

RR, response rate; DCR, disease control rate; PFS, progression-free survival; OS, overall survival; Na+, serum sodium; CI, confidence interval.

### 3.3 Univariate and multivariate survival analyses

In the univariate analysis, features associated with PFS were previous nephrectomy surgery (HR 0.5, 95% CI 0.36–0.70, *p* < 0.01), ECOG-PS ≥1 (HR 1.58, 95% CI 1.24–2.03, *p* < 0.01), IMDC intermediate–poor risk score (HR 1.69, 95% CI 1.29–2.21, *p* < 0.01), and sodium serum ≥140 mEq/L at baseline (HR 0.69, 95% CI 0.53–0.88, *p* < 0.01), at first evaluation (HR 0.71, 95% CI 0.55–0.91, *p* < 0.01), and in both cases (HR 0.66, 95% CI 0.51–0.85, *p* < 0.01). Previous surgery (HR 0.27, 95% CI 0.18–0.40, *p* < 0.01), ECOG-PS ≥1 (HR 2.41, 95% CI 1.75–3.32, *p* < 0.01), IMDC intermediate–poor risk score (HR 1.88, 95% CI 1.32–2.67, *p* < 0.01), and sodium serum ≥140 mEq/L at first evaluation (HR 0.69, 95% CI 0.50–0.94, *p* = 0.02) and both at baseline and first evaluation (HR 0.74, 95% CI 0.54–0.98, *p* = 0.03) were associated with OS (**Table 3**). Multivariate analysis revealed that all items maintained a statistically significant association with PFS and OS (**Table 4**).

**Table 3:** Univariate analysis for PFS and OS.

	HR	95% CI	*p*
**Progression-free survival**			
**Age**			
>70	0.92	0.72–1.19	0.5
**Gender**			
Male	1.05	0.80–1.38	0.7
**Histology**			
Clear cell RCC	0.77	0.53–1.10	0.1
**Previous nephrectomy**			
Yes	**0.50**	**0.36–0.70**	**<0.01**
**ECOG**			
≥1	**1.58**	**1.24–2.03**	**<0.01**
**IMDC score**			
Intermediate–poor	**1.69**	**1.29–2.21**	**<0.01**
**Number of metastatic sites**			
≥3	1.06	0.81–1.38	0.6
**Pre-treatment Na+**			
Na+ ≥140 mEq/L	**0.69**	**0.53–0.88**	**<0.01**
**First evaluation Na+**			
Na+ ≥140 mEq/L	**0.71**	**0.55–0.91**	**<0.01**
**Pre- and first Na+**			
Na+ ≥140 mEq/L	**0.66**	**0.51–0.85**	**<0.01**
**Overall survival**			
**Age**			
>70	1.13	0.83–1.55	0.4
**Gender**			
Male	0.99	0.70–1.40	0.9
**Histology**			
Clear cell RCC	0.88	0.55–1.41	0.6
**Previous surgery**			
Yes	**0.27**	**0.18–0.40**	**<0.01**
**ECOG**			
≥1	**2.41**	**1.75–3.32**	**<0.01**
**IMDC score**			
Intermediate–poor	**1.88**	**1.32–2.67**	**<0.01**
**Number of metastatic sites**			
≥3	0.91	0.64–1.28	0.5
**Line of therapy after TKI**			
>1	1.08	0.79–1.50	0.5
**Pre-treatment Na+**			
Na+ ≥140 mEq/L	0.84	0.61–1.15	0.2
**First evaluation Na+**			
Na+ ≥140 mEq/L	**0.69**	**0.50–0.94**	**0.02**
**Pre- and first Na+**			
Na+ ≥140 mEq/L	**0.74**	**0.54–0.98**	**0.03**

RCC, renal cell carcinoma; IMDC, international metastatic renal cell carcinoma database consortium; TKI, tyrosine kinase inhibitor; HR, hazard ratio; ECOG, Eastern Cooperative Oncology Group; Na, sodium; CI, confidence interval; p, p-value. Bold means statistically significant values.

**Table 4:** Multivariate analysis for PFS and OS.

	HR	95% CI	*p*
**Progression-free survival**			
**Previous surgery**			
Yes	**0.61**	**0.43–0.87**	**<0.01**
**ECOG**			
≥1	**1.42**	**1.10–1.83**	**<0.01**
**IMDC score**			
Intermediate–poor	**1.48**	**1.12–1.95**	**<0.01**
**Pre-treatment Na+**			
Na+ ≥140 mEq/L	**0.77**	**0.60–0.99**	**0.05**
**First evaluation Na+**			
Na+ ≥140 mEq/L	**0.75**	**0.58–0.96**	**0.02**
**Pre- and first Na+**			
Na+ ≥140 mEq/L	**0.71**	**0.55–0.91**	**<0.01**
**Overall survival**			
**Previous surgery**			
Yes	**0.34**	**0.23–0.51**	**<0.01**
**ECOG**			
≥1	**2.10**	**1.52–2.91**	**<0.01**
**IMDC score**			
Intermediate–poor	**1.51**	**1.05–2.17**	**0.02**
**First evaluation Na+**			
Na+ ≥140 mEq/L	**0.77**	**0.56–0.97**	**0.02**
Pre- and first Na+			
Na+ ≥140 mEq/L	**0.81**	**0.59–0.98**	**0.04**

RCC, renal cell carcinoma; IMDC, international Metastatic Renal Cell Carcinoma Database Consortium; ECOG, Eastern Cooperative Oncology Group; HR, hazard ratio; Na, sodium; CI confidence interval; p, p-value. Bold means statistically significant values.

### 3.4 Subgroup analysis according to IMDC risk score

Best response, PFS, and OS were evaluated in a subgroup analysis according to the IMDC risk classification: favorable (*n* = 106) or intermediate/poor (*n* = 211) risk group. In the favorable IMDC risk group, patients with sodium ≥140 mEq/L at first evaluation had statistically significantly higher PFS (31 vs. 16 months, *p* = 0.03) and OS (85 vs. 63 months, *p* = 0.01) compared with patients with natremia <140 mEq/L. Similarly, patients with favorable risk and sodium ≥140 mEq/L both at basal and first assessment had better median PFS (45 vs. 18 months, *p* < 0.01) and median OS (85 vs. 73 months, *p* = 0.03) than patients with sodium values of <140 mEq/L. At pre-treatment evaluation, only median PFS was significantly longer in patients with sodium ≥140 mEq/L (31 vs. 18 months, *p* = 0.01). No differences were observed for RR and DCR in patients with favorable IMDC risk (**Table 5**). In the subgroup analysis regarding IMDC intermediate/poor risk patients, no statistically significant differences were recorded for RR, DCR, PFS, and OS between patients with serum sodium ≥140 mEq/L and patients with serum sodium <140 mEq/L both at baseline and first evaluation (**Table S4**).

**Table 5:** Best response, PFS, and OS NA+ values in favorable risk patients.

	RR, *n* (%)	DCR, *n* (%)	PFS months (95% CI)	OS months (95% CI)
All patients (*n* = 106)	50 (47.1)	96 (90.5)	22 (17–32)	76 (66–NR)
**Pre-treatment Na+,** (*n*) ≥140 mEq/L (69) <140 mEq/L (37)	31 (44.9) 19 (51.3) *p* = 0.5	65 (94.2) 31 (83.8) *p* = 0.1	31 (18–49) 18 (10–24) *p* = 0.01	79 (64–NR) 73 (40–NR) *p* = 0.4
**First evaluation Na+,** (*n*) ≥140 mEq/L (65) <140 mEq/L (41)	29 (44.6) 21 (51.2) *p* = 0.5	58 (89.2) 38 (92.6) *p* = 0.5	31 (19–49) 16 (12–24) *p* = 0.03	85 (73–not reached) 63 (34–76) *p* = 0.01
**Pre- and first Na+,** (*n*) ≥140 mEq/L (50) <140 mEq/L (56)	20 (40.0) 30 (53.5) *p* = 0.2	46 (92.0) 50 (89.2) *p* = 0.6	45 (21–53) 18 (12–23) *p* < 0.01	85 (66–NR) 73 (46–NR) *p* = 0.03

RR, response rate; RC, response complete; PR, partial response; SD, stable disease; PFS, progression-free survival; OS, overall survival; Na, sodium; NR, not reached.

## 4 Discussion

The therapeutic scenario of mRCC is constantly changing, and the prognosis of patients has significantly improved following the introduction of combination therapies (19). Patient stratification into different risk classes remains essential for the treatment choice, but the identification of new prognostic factors is required to choose the best treatment.

To the best of our knowledge, this is the first study to evaluate the effect of normal sodium levels on mRCC outcomes in patients receiving targeted therapy as first-line treatment. A value of baseline serum sodium ≥140 mEq/L, within normal limits, was associated with a significant improvement in PFS, whereas longer PFS and OS were observed in patients with natremia ≥140 mEq/L at first evaluation after the start of treatment. Moreover, in patients with serum sodium ≥140 mEq/L, both at baseline and first evaluation, PFS and OS were found to be longer compared to those with a sodium value of <140 mEq/L.

Previous studies have reported the impact of hyponatremia on the effectiveness of outcomes in patients with mRCC, and normal range values also seem to be correlated with a worse prognosis (**Table 6**). Indeed, in the study by Kawashima et al., a value of serum sodium <138 mEq/L appears to be a significant predictive factor for cancer-specific survival (CSS) in mRCC patients treated with molecular targeted therapy as first-line treatment (21). These authors stratified patients into three groups, namely, severe hyponatremia (≤134 mEq/L), mild hyponatremia (135–137 mEq/L), and normal natremia (≥138 mEq/L), according to a commonly used cutoff value of hyponatremia (serum sodium ≤ 134 mEq/L) (22) and a less frequent and more conservative definition of hyponatremia by Kumar et al. (serum sodium ≤ 137 mEq/L) (23).

**Table 6:** Previous studies correlating serum sodium values with survival in RCC patients.

Author, year (ref)	Setting	No. of patients	Type of treatment	Serum sodium values	Time of evaluation	Efficacy outcome	Response rate
**Vasudev et al., 2008** (14)	Preoperative	220	Surgery	<139 vs. ≥139 mmol/L	Preoperative	Five-year OS: 44.3% vs. 67.6%	–
**Jeppesen et al., 2010** (16)	Metastatic	120	Low-dose and IL2INF-α	<136 vs. ≥136 mM	Baseline	mOS: 5.5 vs. 18.6 months	Lack of response in the hyponatremia group
**Akaza et al., 2011** (20)	Metastatic	42	INF-α and IL2	<135 or ≥135 mmol/L	Baseline	Median survival time at 24 months: 12.2 months vs. NR	Reduced response in the hyponatremia group CR/PR vs. NC/PD: *p* = 0.035 CR/PR/NC vs. PD: *p* = 0.020
**Kawashima et al., 2012** (21)	Metastatic	87	Sorafenib or sunitinib	Severe hyponatremia, ≤134 mEq/L Mild hyponatremia, 135–137 mEq/L Normal natremia, ≥38 mEq/L	Baseline	CSS: 4.2 vs. 9.3 vs. 32.6 months	–
**Schutz et al., 2014** (15)	Metastatic	1,661	Target therapy VEGF or mTOR	<135 vs. ≥135 mmol/L	Baseline	OS: 7.0 vs. 20.9 months	Reduced response in the hyponatremia group TTF: 2.9 vs. 7.4 months, DCR: 54.9% vs. 78.8%

RCC, renal cell carcinoma; CR, complete response; PR, partial response; NC, no change; PD, progression disease; OS, overall survival; PFS, progression-free survival; TTF, time to treatment failure; DCR, disease control rate; IL-2, interleukin-2; INF-α, interferon-α; VEGF, vascular endothelial growth factor; mTOR, mammalian target of rapamycin; CSS, cancer-specific survival.

A higher median CSS time of 32.6 months was observed in patients with natremia ≥138 mEq/L compared with 4.2 and 9.3 months in patients with natremia ≤134 mEq/L and between 135 and 137 mEq/L (*p* < 0.001; normal range), respectively. Serum sodium is frequently measured in clinical practice, but its prognostic value is not clearly defined. Indeed, it is a common electrolyte disorder in hospitalized patients (24, 25) and a negative prognostic factor in several diseases such as liver cirrhosis, congestive heart failure, infections, and several malignancies (4–7, 9, 12, 26). Previous evidence points to a correlation between hyponatremia and poor prognosis in different tumors, including hepatocellular carcinoma, gastric cancer, and localized or metastatic RCC (10, 11, 16).

The prognostic role of preoperative serum sodium in localized RCC patients was first reported in 2008 (14). In this study, a median serum sodium <139 mmol/L (range 135–145 mmol/L) was associated with reduced OS and disease-free survival (14). Subsequently, the impact of serum sodium has also been investigated in patients with metastatic disease. Jeppesen et al. reported low baseline serum sodium (<136 mmol) as a prognostic factor for lower OS (median 5.5 vs. 18.6 months in patients with normonatremia) and a predictive factor for the lack of response in a cohort of 120 patients with mRCC receiving low-dose interleukin-2 and interferon-α (16). Another study confirmed the predictive and prognostic role of a lower-than-normal serum sodium value. The median 2-year OS was 12.2 months for patients with low sodium levels and “not reached” in patients with normal sodium levels (20).

In a large series of 1,661 mRCC patients receiving VEGF or mTOR targeted therapy as first-line treatment, hyponatremia (<135 mmol/L) was associated with a significantly lower OS (7.0 vs. 20.9 months), time to treatment failure (2.9 vs. 7.4 months), and DCR (54.9% vs. 78.8%) compared with normonatremia (*p* < 0.0001 for all comparisons) (15). Only two studies have reported the impact of natremia in different risk categories (15, 21).

Kawashima et al. observed that in patients with intermediate and poor risk, as determined by the MSKCC classification, natremia <138 mEq/L correlated with a significantly worse prognosis compared to natremia ≥138 mEq/L (*p* = 0.05 and *p* = 0.004, respectively) (21). Subsequently, a subgroup analysis by Schutz et al. showed that patients with hyponatremia in the intermediate or poor risk group (according to the IMDC risk classification) had a shorter median OS than patients with normal sodium levels (10.9 vs. 23.5 months and 5.1 vs. 10 months, respectively) (*p* < 0.0001). No significant differences in OS were seen in the favorable risk group with or without hyponatremia (24.3 vs. 41.1 months, respectively; *p* = 0.826) (15).

Contrariwise, we found a correlation between serum sodium <140 mEq/L and statistically significant worse OS and/or PFS in the favorable IMDC risk group compared with patients with ≥140 mEq/L. In IMDC intermediate/poor risk patients, there was no significant difference in PFS and OS between patients with serum sodium ≥140 mEq/L and patients with serum sodium <140 mEq/L. Decreased sodium levels, though within the normal range, may have a lower impact on the prognosis of intermediate or poor risk patients than hyponatremia, but could better discriminate patients with favorable risk.

As reported by Berghmans et al., the incidence of hyponatremia in patients with different types of cancer is around 3.7% (27), although according to Schutz and colleagues, the incidence in mRCC patients appears to be much higher (about 15%) (15).

Regarding causes, hyponatremia usually occurs in cancer patients due to the syndrome of inappropriate ADH release, a paraneoplastic syndrome very frequent in some tumors including small cell lung cancer, head and neck cancers, and breast cancer but not reported in RCC (28). A disturbance of the renin–angiotensin–aldosterone axis, poor function of the adrenal glands, and mild renal impairment due to nephrectomy are considered possible alternatives (14, 29, 30). Alteration of natremia values can also be a consequence of cancer therapy or its side effects, such as diarrhea and vomiting, but the causality needs to be clarified (31). Regardless of the causes, hyponatremia could be considered a warning sign and an indicator of poor prognosis in cancer. Although our results show no relationship between sodium values and response to target therapy, previous studies have reported greater primary refractoriness to targeted therapy or lower tolerance in patients with baseline hyponatremia (15). The influence of serum sodium on mRCC patient survival and treatment response needs to be defined, including real-world cases treated with immune checkpoint inhibitors (32). Furthermore, since lower serum sodium concentration has been associated with higher mortality and worse prognosis in mRCC patients (14, 15), it could be an indicator for therapy choice, specifically for combination therapies. Contrariwise, a higher serum sodium level (in our case ≥140 mEq/L), particularly in patients with IMDC favorable risk, could be an additional positive prognostic factor determining a good response to monotherapy with target therapy. In fact, the choice of treatment with the advent of new drugs is becoming challenging and the classification systems available may not optimally discriminate patients with a favorable prognosis, who could benefit from monotherapy.

This study has some limitations, primarily its retrospective nature and the use of drugs no longer considered the standard of care, excluding selected cases with absolute contraindications to immunotherapy or combined therapy. Secondly, not all patient comorbidities and their related specific concomitant medications were consistently assessed, in particular those regarding antihypertensive drugs. The main strengths of our study are the large number of enrolled patients receiving target therapy as first-line treatment, the evaluation of natremia at baseline and after initiation of treatment, and the adjustment of results for the IMDC prognostic risk criteria.

## 5 Conclusion

In conclusion, we found that in mRCC patients receiving targeted therapy at the forefront, baseline serum sodium <140 mEq/L is associated with a poorer PFS compared with natremia ≥140 mEq/L, while serum sodium <140 mEq/L at first assessment from the initiation of treatment is correlated with worse PFS and OS. Patients with a sodium value of <140 mEq/L at baseline and first assessment have worse OS and PFS than patients with both values >140 mEq/L. A lower, but within the range, sodium concentration (≥135 and ≤145 mEq/L) may be an important factor associated with poorer survival in RCC patients, suggesting its possible use as an additional prognostic variable aside from the well-known nomograms for risk stratification.

## Data Availability Statement

The raw data supporting the conclusions of this article will be made available by the authors, without undue reservation.

## Ethics statement

This study was reviewed and approved by Tuscany section AREA VASTA CENTRO, number:14565_oss. The patients/participants provided their written informed consent to participate in this study.

## Author contributions

GR had full access to all the data in the study and takes responsibility for the integrity of the data and the accuracy of the data analysis. Study concept and design: GR. Acquisition of data: EG, CO, MC, and LD. Recruitment of patients: UD, MM, GP, PS, NB, MS, LG, and RC. Analysis and interpretation of data: GR. Drafting of the manuscript: GR and LA. Critical revision of the manuscript for important intellectual content: GR, LA, UD, and SB. Statistical analysis: GR. Supervision: LA. All authors contributed to the article and approved the submitted version.

## Conflict of interest

The authors declare that the research was conducted in the absence of any commercial or financial relationships that could be construed as a potential conflict of interest.

## Publisher’s note

All claims expressed in this article are solely those of the authors and do not necessarily represent those of their affiliated organizations, or those of the publisher, the editors and the reviewers. Any product that may be evaluated in this article, or claim that may be made by its manufacturer, is not guaranteed or endorsed by the publisher.
