# Supplementary material for: Prognostic value of normal sodium levels in patients with metastatic renal cell carcinoma receiving tyrosine kinase inhibitors
Source: Front Oncol. 2022 Aug 16;12:918413. doi: 10.3389/fonc.2022.918413 (PMC9424544; doi:10.3389/fonc.2022.918413)
Supplement: Supplementary file 1 [file Table_1.docx]

**Table S1.** Patients’ baseline characteristics according pre-treatment Na+.

|  | ≥140 mEq/L  185 (58.3%) | <140 mEq/L  132 (41.7%) | *p* |
| --- | --- | --- | --- |
| Age  Median (range) | 67 (36-86) | 66 (37-89) | 0.2 |
| Gender, n (%)  Male | 128 (69.1) | 104 (78.8) | 0.1 |
| Histology, n (%)  Clear-cell RCC | 161 (87.0) | 114 (86.3) | 0.9 |
| Previous surgery, n (%)  Yes | 164 (88.6) | 107 (81.0) | 0.1 |
| ECOG, n (%)  ≥1 | 86 (46.4) | 66 (50.0) | 0.5 |
| IMDC score, n (%)  Intermediate-poor | 116 (62.7) | 95 (71.9) | 0.1 |
| Number of metastatic sites, n (%)  ≥3 | 50 (27.0) | 45 (34.0) | 0.2 |
| First-Line Therapy, n (%)  Sunitinib  Pazopanib  Cabozantinib | 99 (53.5%  68 (36.7)  18 (9.8) | 75 (56.8)  42 (31.8)  15 (11.4) | 0.6 |
| Line of therapy after TKI, n (%)  >1 | 49 (26.4) | 48 (36.3) | 0.1 |

*RCC: renal cell carcinoma, ECOG: eastern cooperative oncology group; IMDC: [international metastatic renal cell carcinoma database consortium](https://www.imdconline.com/); TKI: tyrosine kinase inhibitor; N: serum sodium; P: p value.*

|  | ≥140 mEq/L  184 (58.0%) | <140 mEq/L  133 (42.0%%) | *p* |
| --- | --- | --- | --- |
| Age  Median (range) | 66 (36-86) | 69 (37-89) | 0.1 |
| Gender  Male | 132 (71.7) | 100 (75.1) | 0.5 |
| Histology  Clear-cell RCC | 162 (88.0) | 113 (84.9) | 0.4 |
| Previous surgery  Yes | 162 (88.0) | 109 (81.9) | 0.1 |
| ECOG  ≥1 | 84 (45.6) | 68 (51.1) | 0.3 |
| IMDC score  Intermediate-poor | 119 (64.6) | 92 (69.1) | 0.4 |
| Number of metastatic sites  ≥3 | 53 (28.8) | 42 (31.5) | 0.5 |
| First-Line Therapy  Sunitinib  Pazopanib  Cabozantinib | 100 (54.3)  66 (35.9)  18 (9.8) | 74 (55.6)  44 (33.1)  15 (11.3) | 0.8 |
| Line of therapy after TKI  >1 | 53 (28.8) | 44 (33.0) | 0.4 |

**Table S2.** Patients’ baseline characteristics according first evaluation Na+.

*RCC: renal cell carcinoma, ECOG: eastern cooperative oncology group; IMDC: [international metastatic renal cell carcinoma database consortium](https://www.imdconline.com/); TKI: tyrosine kinase inhibitor; N: serum sodium; P: p value.*

**Table S3.** Patients’ baseline characteristics according pre-and first assessment Na+.

|  | ≥140 mEq/L  139 (43.8%) | <140 mEq/L  178 (56.2%) | *p* |
| --- | --- | --- | --- |
| Age  Median (range) | 66  36-86 | 67  37-89 | 0.6 |
| Gender, n (%)  Male | 96 (69.0) | 136 (76.4) | 0.1 |
| Histology, n (%)  Clear-cell RCC | 122 (87.7) | 153 (85.9) | 0.5 |
| Previous surgery, n (%)  Yes | 123 (88.4) | 148 (83.1) | 0.2 |
| ECOG, n (%)  ≥1 | 63 (45.3) | 89 (50.0) | 0.4 |
| IMDC score, n (%)  Intermediate-poor | 89 (64.0) | 122 (68.5) | 0.4 |
| Number of metastatic sites, n (%)  ≥3 | 37 (26.6) | 58 (32.5) | 0.2 |
| First-Line Therapy, n (%)  Sunitinib  Pazopanib  Cabozantinib | 77 (55.4)  48 (34.5)  14 (10.0) | 97 (54.4)  62 (34.8)  19 (10.6) | 0.9 |
| Line of therapy after TKI, n (%)  >1 | 40 (28.7) | 57 (32.0) | 0.5 |

*RCC: renal cell carcinoma, ECOG: eastern cooperative oncology group; IMDC: [international metastatic renal cell carcinoma database consortium](https://www.imdconline.com/); TKI: tyrosine kinase inhibitor; N: serum sodium; P: p value.*

**Table S4:** Best response, PFS and OS according to NA+ values in intermediate/poor risk patients.

|  | RR  n (%) | DCR  n (%) | PFS M-months  (95% IC) | OS M-months  (95% IC) |
| --- | --- | --- | --- | --- |
| All patients  (n=211) | 78 (36.9) | 168 (79.6) | 11  (9-13) | 43  (29-52) |
| Pre-treatment Na+, (n)  ≥140 mEq/L (116)  <140 mEq/L (95) | 40 (34.4)  38 (40.0)  *p*=0.4 | 94 (81.0)  74 (77.8)  *p*=0.6 | 12 (9-17)  10 (7-13)  *p*=0.2 | 43 (24-64)  43 (24-55)  *p*= 0.8 |
| First evaluation Na+, (n)  ≥140 mEq/L (119)  <140 mEq/L (92) | 45 (37.8)  33 (35.8)  *p*=0.7 | 100 (84.0)  68 (73.9)  *p*=0.1 | 12 (9-14)  9 (6-13)  *p*=0.1 | 49 (33-71)  35 (23-46)  *p*=0.3 |
| Pre-and first Na+, (n)  ≥140 mEq/L (89)  <140 mEq/L (122) | 31 (34.8)  47 (38.5)  *p*=0.5 | 75 (84.2)  93 (76.2)  *p*=0.1 | 12 (9-19)  10 (7-13)  *p*=0.1 | 49 (33-78)  36 (23-46)  *p*=0.3 |

*RR: response rate; RC: response complete; PR: prtial response; SD: stable disease; PFS: progression free survival; OS: overall survival; Na: sodium.*
